# Efficacy of Transvaginal Surgery Using an ORIHIME Mesh With Wider Arms and Adjusted Length

**DOI:** 10.7759/cureus.57106

**Published:** 2024-03-28

**Authors:** Kenji Kuroda, Koetsu Hamamoto, Kazuki Kawamura, Hiroaki Kobayashi, Akio Horiguchi, Keiichi Ito

**Affiliations:** 1 Department of Urology, National Defense Medical College, Tokorozawa, Saitama, JPN

**Keywords:** longitudinal mesh length, mesh arm width, transvaginal mesh surgery, female urology, pelvic organ prolapse

## Abstract

Introduction: Transvaginal mesh surgery (TVM) is an effective treatment measure for pelvic organ prolapse (POP). However, the ORIHIME mesh (Kono Seisakusho, Japan, Tokyo), which has the disadvantage of poor adherence to tissues, is currently the only product available for this procedure. Our research team has previously developed a modified ORIHIME mesh with wider arms with the aim of minimizing the risk of POP recurrence. Additionally, the length of the mesh behind the anterior vaginal wall has been adjusted to prevent urinary incontinence. The current study aims to examine the efficacy of this modified mesh in minimizing postoperative complications in patients undergoing uphold-type TVM.

Methods: The data of 84 patients who underwent TVM using ORIHIME at our hospital since July 2019 were retrospectively analyzed. The patients were divided into three groups as follows: (a) normal arms (NA; arm width < 6 cm; n = 29 cases); (b) wide arms without length adjustment (WA and LA (-); arm width > 6 cm; n = 27 cases); and (c) wide arms with length adjustment (WA and LA (+); n = 28 cases). Data were collected using various questionnaires, and the residual urine volume was measured before and after surgery. Additionally, the 60-minute pad test was performed where possible, and the recurrence and complication rates were recorded.

Results: The incidence of mesh exposure and urinary incontinence in daily life tended to be lower in the WA and LA (+) groups, although this difference was not statistically significant. The one-year postoperative POP recurrence rate, residual urine volume, International Prostate Symptom Score (IPSS), Overactive Bladder Symptom Score (OABSS), and the International Consultation on Incontinence Questionnaire-Short Form (ICIQ-SF) score were significantly lower in the WA and LA (+) groups compared to the other groups.

Conclusion: Uphold-type TVM using the modified ORIHIME mesh with wider arms and adjusted length was associated with better postoperative treatment outcomes compared to TVM using the traditional ORIHIME mesh.

## Introduction

Surgical meshes were initially designed for use in hernia repair [1]. However, since the 1990s, gynecological surgeons have utilized these materials for transvaginal mesh (TVM) surgical treatment of pelvic organ prolapse (POP) and stress urinary incontinence (SUI) [2]. The surgical outcomes of TVM in patients with POP have been found to be significantly superior to that of native tissue repair, with the one-year post-surgical POP recurrence rates of the former being approximately 10% [3].

Polyform (Boston Scientific Japan), a synthetic polypropylene (PP) soft mesh, was banned for TVM use in Japan in April 2019 because of the increasing incidence of mesh-related postoperative complications in some countries (excluding Japan). The use of ORIHIME, a polytetrafluoroethylene (PTFE) mesh, was subsequently approved, and this is currently the only mesh product available for TVM in Japan. Previous studies comparing the postoperative clinical outcomes and quality of life of patients undergoing TVM surgery using PP or PTFE reported no significant differences in short-term outcomes between the two materials, with the latter being identified as one of the most acceptable mesh materials for pelvic floor reconstruction [4,5].

However, the differences between these materials and other mesh products necessitated an investigation of the role of mesh shapes in treatment outcomes. Therefore, our research team previously developed a modified ORIHIME mesh with wider arms and demonstrated that it effectively reduced postoperative recurrence rates in patients treated with uphold-type TVM using wide-arm ORIHIME [6]. Evidence also suggests that TVM patients exhibited a higher risk of de novo urinary incontinence compared to those treated with native tissue repair or sacrocolpopexy [7-10], with one study suggesting that this could potentially be attributed to excessive traction behind the anterior vaginal wall, leading to unnecessary opening of the bladder neck [11].

Based on these findings, our research team adjusted the length of the modified ORIHIME mesh behind the anterior vaginal wall, with the aim of minimizing the risk of postoperative de novo urinary incontinence. Therefore, the current study compared the treatment outcomes and efficacy of uphold-type TVM by mesh shape (i.e., modified mesh shape with wider arms and adjusted length vs other mesh shapes).

## Materials and methods

Patients

Eighty-four patients with POP treated with TVM at our hospital since July 2019 were enrolled in this study. The surgical indication was POP of stage ≥ 3 with symptoms such as a feeling of vaginal prolapse. All procedures performed in this study were in accordance with the ethical standards of the National Defense Medical College (Saitama, Japan; ID 4219). This study protocol was accepted on August 21, 2020, by the National Defense Medical College Ethics Committee. Written informed consents were collected from all patients. All patients who underwent TVM during this period were considered eligible for inclusion in this study, and those who did not give consent to participate were excluded. This study is a retrospective study design consisting of an evaluation of past medical records.

Surgical methods and groups

All patients underwent the uphold-type TVM that was primarily carried out in accordance with the techniques reported by our previous report. Additionally, the ORIHIME (Kono Seisakusho, Japan, Tokyo) mesh and other surgical equipment were used in the previous study [6]. Vaginal hysterectomy was not performed in any cases. Since this is a retrospective study, no randomization was performed.

**Figure 1:**
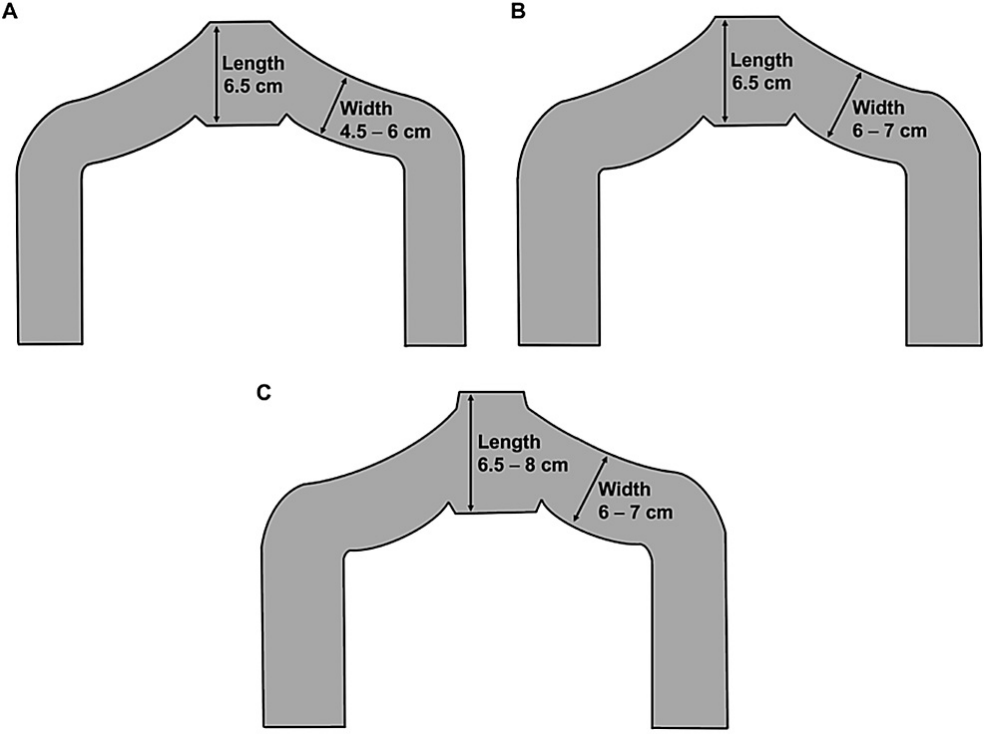
The stencil papers used for the exteriorized arms Stencil papers for normal arm mesh (NA) (A), wide arms (WA) without length adjustment (LA) (WA and LA (-)) (B), wide arms plus length adjustment (WA and LA (+)) (C). These figures were created by the first author, Kuroda using Procreate (Savage Interactive Pty., Hobart, Australia). Image credit: Kenji Kuroda

The meshes were cut to fit each stencil paper with two arms in advance. The arm width was initially set to 4.5-6 cm but later increased to approximately 6-7 cm along with adjustment of mesh length behind the anterior vaginal wall (Figure 1). The patients were divided into three groups based on mesh shape used as follows: (a) normal arm (NA; arm width < 6 cm; n = 29 cases); (b) wide arms without length adjustment (WA and LA (-); arm width > 6 cm; n = 27 cases); and (c) wide arms with length adjustment (WA and LA (+); n = 28 cases). The mesh length was determined by measuring the distance between the cervix or vaginal vault (grasped using Allis forceps) and point Aa (i.e., 3 cm medial to the bladder neck) and then adjusted such that it did not pull the bladder neck excessively in a cephalad direction (Figure 1).

The median postoperative observation period was 12.3 (range: 12.0-54.9) months. The median (minimum-maximum) surgical durations for the NA and WA and LA (-) groups were 1.0 (0.6-1.8) and 1.2 (0.8-1.4) hours, respectively, while the mean (± standard deviation) duration in the WA and LA (+) group was 1.0 (± 0.2) hours.

Assessment methods for preoperative and postoperative parameters

To assess the change in the quality of life (QoL) at one year postoperatively, residual urine volume, 60-min pad test, International Prostate Symptom Score (IPSS), Overactive Bladder Symptom Score (OABSS), and International Consultation on Incontinence Questionnaire-Short Form (ICIQ-SF) were used. ICIQ-SF is available by logging in to the ICIQ Group website and agreeing to the terms and conditions (https://iciq.net/register).

Prolapse recurrence was defined as the most dependent portion being at the pelvic organ prolapse questionnaire (POP-Q) stage equal to or greater than 2, which means that the most distal prolapse portion is 1 cm from the hymen plane, according to our previous report [6]. Urinary incontinence was defined as a daily life disturbance with incontinence. Mesh exposure was vaginally and/or visually examined using a vaginal scope.

Statistical analysis

The Steel-Dwass test and Student's t-test were used to compare preoperative values among the three groups. Wilcoxon’s signed-rank test was used to compare preoperative and 3-, 6-, and 12-month postoperative values within each group, whereas Pearson’s chi-square test was used to compare preoperative and one-year postoperative outcomes by the mesh type and TVM method. Statistical analysis was performed using JMP PRO (version 17; SAS Institute, Cary, NC). A p value < 0.05 was considered statistically significant.

## Results

Table 1 shows the clinical data, such as age, BMI, POP-Q stage, details of POP, previous laparotomies, blood loss, and operative time, and major complications. No serious intraoperative or postoperative complications were observed.

**Table 1:** Clinical characteristics of the patients LA: length adjustment. BMI: body mass index. POP-Q: pelvic organ prolapse quantification. VVP: vaginal vault prolapse *Steel-Dwass test was used. **Student's t-test was used. ***Pearson’s chi-square test was used.

	1. Normal Arm (n = 29)		2. Wide Arm, LA (-) (n = 27)		3. Wide Arm, LA (+) (n = 28)			p value
									Age	79 (55-86)		76.6 ± 7.4		73.4 ± 8.4		1 vs 3	0.0079*
							2 vs 3	0.2594
BMI (kg/m^2^)	23.6 ± 3.4		24.5 ± 3.5		25.7 ± 3.3		1 vs 3	0.0223**
							2 vs 3	0.2131
POP-Q stage	Stage 3 (n = 20)	n	Stage 3 (n = 25)	n	Stage 3 (n = 27)	n	1 vs 3	0.0064***
	Cystocele	0	Cystocele	6	Cystocele	9	2 vs 3	0.5311
	Cystocele + Hysterocele	15	Cystocele + Hysterocele	14	Cystocele + Hysterocele	9		
	Cystocele + VVP	5	Cystocele + VVP	4	Cystocele + VVP	7		
	Hysterocele	0	Hysterocele	1	Hysterocele	2		
	Stage 4 (n = 9)	n	Stage 4 (n = 2)	n	Stage 4 (n = 1)	n		
	Cystocele + Hysterocele	5	Cystocele + Hysterocele	1	Cystocele + Hysterocele	0		
	Cystocele + VVP	4	Cystocele + VVP	1	Cystocele + VVP	1		
Previous laparotomy	1 (0-3)		0 (0-3)		1 (0-4)		1 vs 3	0.9819
							2 vs 3	0.2995
Blood loss (mL)	26 (4-250)		20 (3-89)		21.5 (5-128)		1 vs 3	0.8032
							2 vs 3	0.9302
Operative time (h)	1.0 (0.6-1.8)		1.2 (0.8-1.4)		1.0 ± 0.2		1 vs 3	0.9931
							2 vs 3	0.9719
Complications	0		0		0			

**Figure 2:**
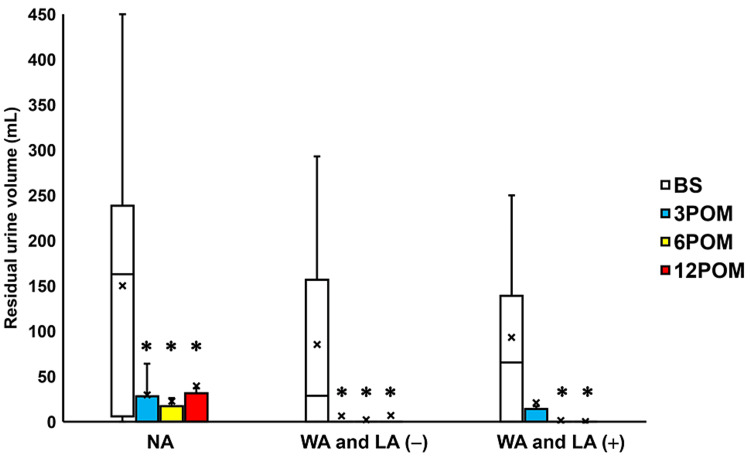
Change in the residual urine volume before and after surgery Box plots showing a significant decrease in residual urine volume 3-12 months after surgery (3-12 POM) in the normal arm (NA) and wide arms without length adjustment (WA and LA (-)) groups, and 6-12 months after surgery in the wide arms with length adjustment (WA and LA (+)) group. * = significantly different from the period before surgery (BS) (all p < 0.05). NA: normal arms, WA: wide arms, LA: length adjustment

**Figure 3:**
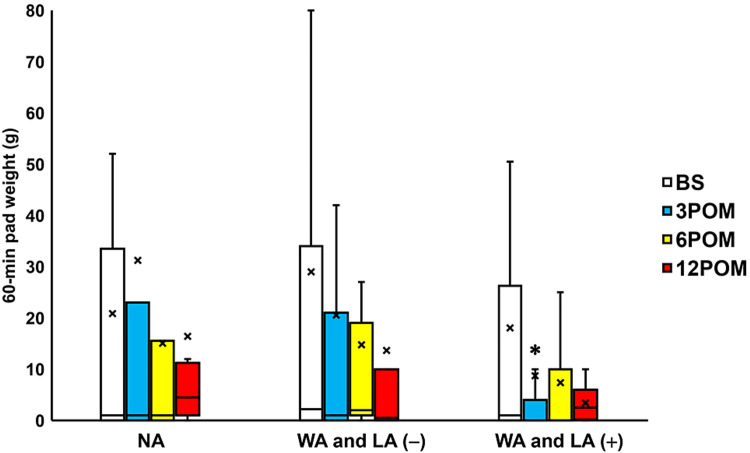
Change in the 60-min pad weight test before and after surgery Box plots showing a significant change in the 60-min pad weight test three months after surgery (three POM) in the wide arms with length adjustment (WA and LA (+)) group. * = significantly different from the period before surgery (BS) (p = 0.0469). NA: normal arms, WA: wide arms, LA: length adjustment

Comparison with preoperative values showed that the residual urine volume was significantly decreased three, six, and 12 months after surgery in the NA and WA and LA (-) groups and six and 12 months after surgery in the WA and LA (+) group (all p < 0.05; Figure 2). However, the 60-minute pad weight test findings exhibited a significant change three months postoperatively in the WA and LA (+) group only (p = 0.0469; Figure 3).

**Figure 4:**
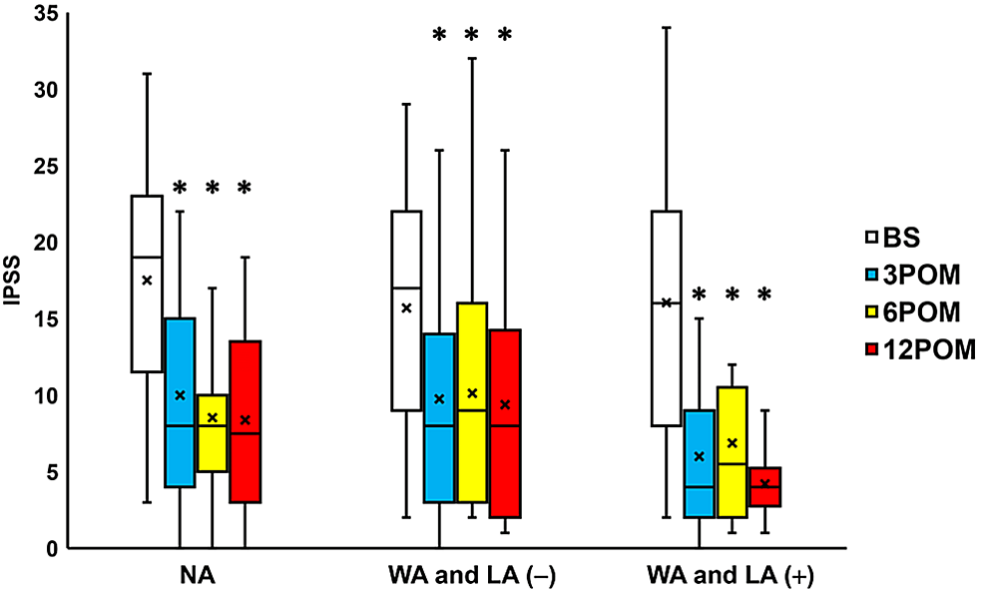
Change in the IPSS before and after surgery Box plots showing a significant change 3-12 months after surgery (3-12 POM) compared to preoperative scores in the IPSS in the normal arm (NA) group, wide arms without length adjustment (WA and LA (-)) group, and the wide arms with length adjustment (WA and LA (+)) group. * = significantly different from the period before surgery (BS) (all p < 0.05). NA: normal arms, WA: wide arms, LA: length adjustment

**Figure 5:**
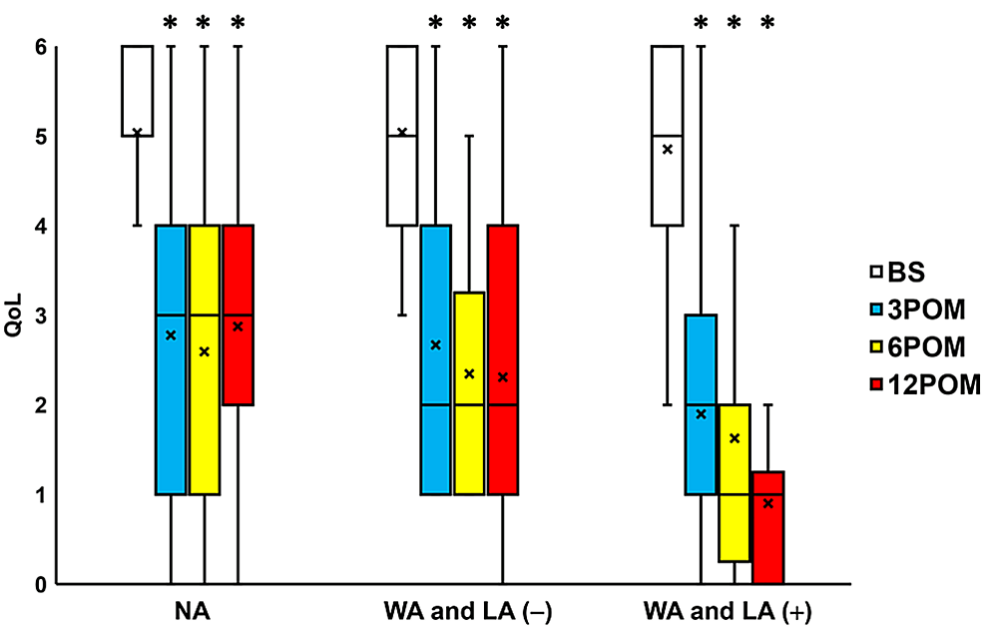
Change in the QoL score before and after surgery Box plots showing a significant change in QoL score 3-12 months after surgery (3-12 POM) in the normal arm (NA) group, wide arms without length adjustment (WA and LA (-)) group, and the wide arms with length adjustment (WA and LA (+)) group. * = significantly different from the period before surgery (BS) (all p < 0.05). NA: normal arms, WA: wide arms, LA: length adjustment

**Figure 6:**
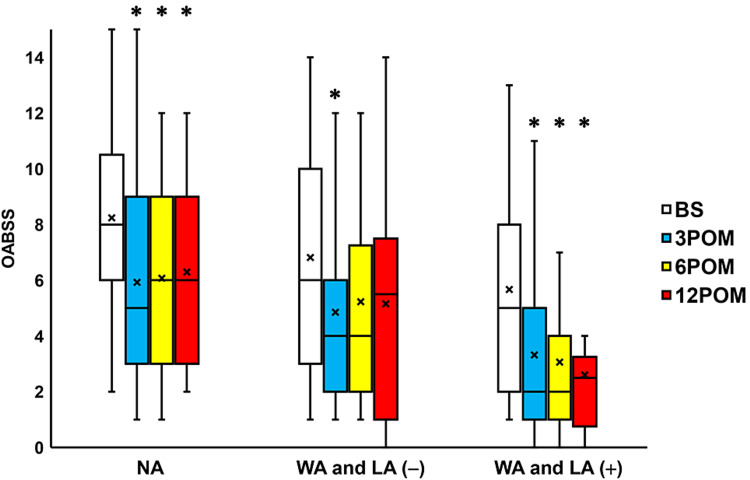
Change in the OABSS before and after surgery Box plots showing a significant improvement for OABSS 3-12 months after surgery (3-12 POM) in the normal arm (NA) group, wide arms with length adjustment (WA and LA (+)) group, and three months after surgery in the wide arms without length adjustment (WA and LA (-)) group. * = significantly different from the period before surgery (BS) (all p < 0.05). NA: normal arms, WA: wide arms, LA: length adjustment

**Figure 7:**
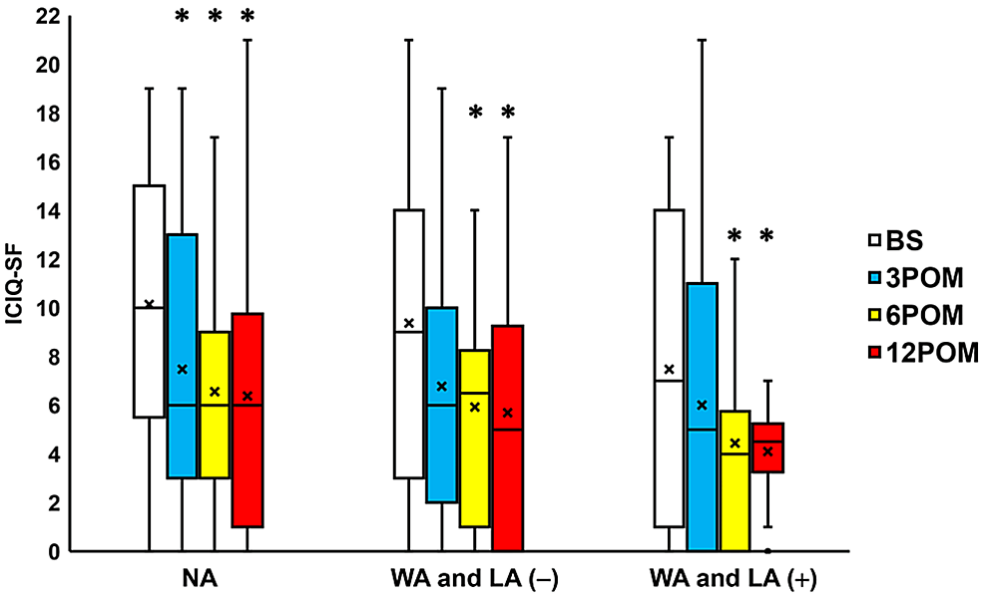
Change in ICIQ-SF before and after surgery Box plots showing a significant improvement in ICIQ-SF found 3-12 months after surgery (3-12 POM) in the normal arm (NA) group and six and 12 months after surgery in the wide arms without length adjustment (WA and LA (-)) group, and the wide arms with length adjustment (WA and LA (+)) group. * = significantly different from the period before surgery (BS) (all p < 0.05). NA: normal arms, WA: wide arms, LA: length adjustment

Compared to the preoperative values, IPSS scores were seen to significantly change three, six, and 12 months after surgery in the NA group, the WA and LA (-) group and the WA and LA (+) groups (all p < 0.05; Figure 4). Moreover, the QoL scores exhibited a significant change three, six, and 12 months after surgery in the NA, WA and LA (-), and WA and LA (+) groups (all p < 0.05; Figure 5). The OABSS improved significantly 3-12 months after surgery in the NA and WA and LA (+) groups (all p < 0.05; Figure 6) and three months after surgery in the WA and LA (-) group (p = 0.0207; Figure 6). The ICIQ-SF scores also decreased significantly 3-12 months after surgery in the NA group and 6-12 months after surgery in the WA and LA (-) and WA and LA (+) groups (all p < 0.05; Figure 7).

No significant differences in the incidence of mesh exposure and urinary incontinence that interfered with daily life were observed between the WA and LA (+) and other groups. However, the prolapse recurrence rates were seen to significantly differ between the WA and LA (+) and NA groups (p = 0.0109; Table 2).

**Table 2:** Association between mesh shapes and the presence of incontinence, mesh exposure, and prolapse recurrence after surgery LA: length adjustment *Pearson’s chi-square test was used.

		1. Normal Arm	2. Wide Arm, LA (-)	3. Wide Arm, LA (+)		p value
Urinary incontinence	Present	6 (20.7%)	5 (18.5%)	3 (10.7%)	1 vs 3	0.3018
	Absent	23 (79.3%)	22 (81.5%)	25 (89.3%)	2 vs 3	0.4118
Mesh exposure	Present	0 (0%)	3 (11.1%)	0 (0%)	1 vs 3	No value
	Absent	29 (100%)	24 (88.9%)	28 (100%)	2 vs 3	0.0697
Prolapse recurrence	Present	6 (20.7%)	1 (3.7%)	0 (0%)	1 vs 3	0.0109*
	Absent	23 (79.3%)	26 (96.3%)	28 (100%)	2 vs 3	0.3041

Comparison of preoperative and one-year postoperative outcomes by mesh shape showed no significant differences in the 60-min pad test findings and QoL scores. However, the residual urine volume was seen to be significantly improved in the WA and LA (+) group compared to the NA group (p = 0.0109), while the IPSS total score and OABSS were significantly improved in the WA and LA (+) group compared to the WA and LA (-) group (p = 0.0344 and 0.0073, respectively; Table 2). Moreover, the ICIQ-SF score was significantly improved in the WA and LA (+) group compared to all other groups (p = 0.0109 and 0.0349, respectively; Table 3).

**Table 3:** Association between mesh shapes and postoperative outcomes at one year compared to preoperative conditions LA: length adjustment. IPSS: international prostate symptom score. QoL: quality of life score. OABSS: overactive bladder symptom score. ICIQ-SF: international consultation on incontinence questionnaire-short form. *Pearson’s chi-square test was used.

	1. Normal Arm	2. Wide Arm, LA (-)	3. Wide Arm, LA (+)		p value
Residual urine volume					
Worse	6 (20.7%)	2 (7.4%)	0 (0%)	1 vs 3	0.0109*
Better or no change	23 (79.3%)	25 (92.6%)	28 (100%)	2 vs 3	0.1424
60-min pad test	n=28	n=23	n=12		
Worse	10 (35.7%)	7 (30.4%)	1 (8.3%)	1 vs 3	0.0755
Better or no change	18 (64.3%)	16 (69.6%)	11 (91.7%)	2 vs 3	0.1394
IPSS					
Worse	3 (10.3%)	4 (14.8%)	0 (0%)	1 vs 3	0.0804
Better or no change	26 (89.7%)	23 (85.2%)	28 (100%)	2 vs 3	0.0344*
QoL					
Worse	2 (6.9%)	0 (0%)	0 (0%)	1 vs 3	0.1572
Better or no change	27 (93.1%)	27 (100%)	28 (100%)	2 vs 3	no value
OABSS					
Worse	4 (13.8%)	10 (37.0%)	2 (7.1%)	1 vs 3	0.4134
Better or no change	25 (86.2%)	17 (63.0%)	26 (92.9%)	2 vs 3	0.0073*
ICIQ-SF					
Worse	6 (20.7%)	3 (11.1%)	0 (0%)	1 vs 3	0.0109*
Better or no change	23 (79.3%)	24 (88.9%)	28 (100%)	2 vs 3	0.0349*

## Discussion

The WA and LA (+) group exhibited significantly decreased residual urine volume, 3-12 months after surgery; 60-min pad weight three months postoperatively; and questionnaire scores when compared to the other groups (Figures 2-7). Moreover, the POP recurrence rate was significantly decreased in the WA and LA (+) group compared to the NA group (Table 2). No significant differences in the rates of urinary incontinence and mesh exposure were observed among the three groups. However, the WA and LA (+) group exhibited greater improvement in residual urine volume one year postoperatively compared with the NA group, while the ICIQ-SF scores were significantly improved in the WA and LA (+) group compared with the other groups (Table 3).

The need for the establishment of strategies to minimize POP recurrence is particularly emphasized by the fact that ORIHIME is now the only mesh product available for TVM surgery. Evidence suggests that the ORIHIME mesh exhibits weaker adherence to the surrounding tissues, and the associated increase in recurrence rates can potentially be attributed to the mesh sliding off the fixation site, rather than inadequate anchorage as observed in synthetic polypropylene meshes [4]. A previous study conducted by our research team showed that the modified ORIHIME mesh with wider arms effectively decreased the incidence of postoperative recurrence and other complications [6]. However, this study did not consider the role of adjusted mesh length in decreasing the risk of urinary incontinence and consequent QoL impairment. Therefore, the current study aimed to address this limitation, and the findings confirmed the usefulness of the mesh with wider arms and adjusted length.

In 2019, the US Food and Drug Administration (FDA) mandated that all suppliers providing surgical meshes intended for use during transvaginal repair of anterior vaginal wall prolapse should cease marketing and distribute their products promptly. The number of patients undergoing laparoscopic sacrocolpopexy (LSC) as an alternative to TVM has increased in Japan. Several studies have compared the postoperative outcomes of both procedures in POP patients, with some reporting that a higher proportion of the TVM group exhibited adverse events (7.1% vs 1.8%; p < 0.001) and genitourinary complications (5.7% vs 1.1%; p < 0.001) [12] and others reporting that the LSC group exhibited fewer serious adverse events compared with the TVM group and that both surgical methods exhibited comparable outcomes overall [9,13]. The previous study conducted by our research group concluded that, although unsuitable for patients with stage 4 POP, TVM provided adequate treatment efficacy for patients with POP stages equal to or smaller than 3 [6].

TVM has been shown to be associated with a relatively high incidence of postoperative SUI when compared with LSC or native tissue repair (NTR) [14-16]. Evidence suggests that this could potentially be attributed to the fact that installation of the mesh tends to overcorrect the prolapse, thereby applying more force to the anterior vaginal wall and increasing the risk of SUI [14,17]. A concurrent anterior operation (usually colporrhaphy) that offers softer support for the urethra and bladder neck, thereby lowering the risk of de novo SUI, may be considered as an alternative [14]. Conversely, some studies found no significant differences in SUI incidence rates between patients undergoing TVM and those treated using other surgical methods [9,18]. The lower incidence of incontinence and improved ICIQ-SF observed in the WA and LA (+) group of the current study were consistent with previous evidence [9,18].

The prolapse quality of life questionnaire (P-QOL) is often used to quantify improvement in POP patients [19], although it was not included in the current study as several values were missing. Instead, medical interviews and physical examinations in the lithotomy position were carried out to evaluate the degree of improvement in POP patients. IPSS, in addition to its role in the treatment of males diagnosed with benign prostatic hyperplasia, has been used to evaluate voiding dysfunction in women with POP [20,21].

The current study had several limitations, including a relatively small sample size, unavailability of data on longitudinal treatment outcomes over several years, and a retrospective single-center study design. Nevertheless, within the limitations of this study, it can be concluded that the modified ORIHIME mesh with wider arms and adjusted length represents a viable therapeutic strategy.

## Conclusions

Uphold-type TVM using the modified ORIHIME mesh with wider arms and adjusted length can potentially reduce the risk of post-surgical POP recurrence and urinary incontinence.

Uphold-type TVM surgery with wider arms and length adjustment may become a mainstream treatment for POP in the future. We will continue to follow the results of this procedure carefully.

## References

[1] Usher FC (1959). A new plastic prosthesis for repairing tissue defects of the chest and abdominal wall. Am J Surg.

[2] Mangir N, Aldemir Dikici B, Chapple CR, MacNeil S (2019). Landmarks in vaginal mesh development: polypropylene mesh for treatment of SUI and POP. Nat Rev Urol.

[3] Maher C, Baessler K (2006). Surgical management of anterior vaginal wall prolapse: an evidencebased literature review. Int Urogynecol J Pelvic Floor Dysfunct.

[4] Kuwata T, Watanabe M, Kashihara H, Kato C, Takeyama M (2023). Histopathological considerations of transvaginally implanted polytetrafluoroethylene mesh in human biological tissues. Continence Reports.

[5] Nakai K, Hamuro A, Kitada K (2021). Preliminary evaluation of the short-term outcomes of polytetrafluoroethylene mesh for pelvic organ prolapse. J Obstet Gynaecol Res.

[6] Kuroda K, Hamamoto K, Kawamura K, Masunaga A, Kobayashi H, Horiguchi A, Ito K (2024). Favorable postoperative outcomes after transvaginal mesh surgery using a wide-arm ORIHIME® mesh. Cureus.

[7] Maher C, Feiner B, Baessler K, Christmann-Schmid C, Haya N, Brown J (2016). Surgery for women with apical vaginal prolapse. Cochrane Database Syst Rev.

[8] Kluivers KB, Kamping M, Milani AL, IntHout J, Withagen MI (2023). Subjective outcomes 12 years after transvaginal mesh versus native tissue repair in women with recurrent pelvic organ prolapse; a randomized controlled trial. Int Urogynecol J.

[9] Liu CK, Tsai CP, Chou MM, Shen PS, Chen GD, Hung YC, Hung MJ (2014). A comparative study of laparoscopic sacrocolpopexy and total vaginal mesh procedure using lightweight polypropylene meshes for prolapse repair. Taiwan J Obstet Gynecol.

[10] Kusuda M, Kagami K, Takahashi I, Nozaki T, Sakamoto I (2022). Comparison of transvaginal mesh surgery and robot-assisted sacrocolpopexy for pelvic organ prolapse. BMC Surg.

[11] Nomura Y, Okada Y, Hiramatsu A, Matsubara E, Kato K, Yoshimura Y (2021). A new method of adjusting mesh tension using cystoscopy during laparoscopic sacrocolpopexy. Int Urogynecol J.

[12] Obinata D, Sugihara T, Yasunaga H (2018). Tension-free vaginal mesh surgery versus laparoscopic sacrocolpopexy for pelvic organ prolapse: analysis of perioperative outcomes using a Japanese national inpatient database. Int J Urol.

[13] Li YL, Chang YW, Yang TH, Wu LY, Chuang FC, Kung FT, Huang KH (2020). Mesh-related complications in single-incision transvaginal mesh (TVM) and laparoscopic abdominal sacrocolpopexy (LASC). Taiwan J Obstet Gynecol.

[14] Altman D, Väyrynen T, Engh ME, Axelsen S, Falconer C (2011). Anterior colporrhaphy versus transvaginal mesh for pelvic-organ prolapse. N Engl J Med.

[15] Galan LE, Bartolo S, De Graer C, Delplanque S, Lallemant M, Cosson M (2023). Comparison of early postoperative outcomes for vaginal anterior sacrospinous ligament fixation with or without transvaginal mesh insertion. J Clin Med.

[16] Fu L, Huang G, Sun Z, Zhu L (2023). Predicting the occurrence of stress urinary incontinence after prolapse surgery: a machine learning-based model. Ann Transl Med.

[17] Alas AN, Chinthakanan O, Espaillat L, Plowright L, Davila GW, Aguilar VC (2017). De novo stress urinary incontinence after pelvic organ prolapse surgery in women without occult incontinence. Int Urogynecol J.

[18] Wei D, Wang P, Niu X, Zhao X (2019). Comparison between laparoscopic uterus/sacrocolpopexy and total pelvic floor reconstruction with vaginal mesh for the treatment of pelvic organ prolapse. J Obstet Gynaecol Res.

[19] Digesu GA, Khullar V, Cardozo L, Robinson D, Salvatore S (2005). P-QOL: a validated questionnaire to assess the symptoms and quality of life of women with urogenital prolapse. Int Urogynecol J Pelvic Floor Dysfunct.

[20] Toyama Y, Suzuki Y, Nakayama S, Endo Y, Kondo Y, Ichikawa M, Akira S (2022). Outcome of modified laparoscopic sacrocolpopexy and its effect on voiding dysfunction. J Nippon Med Sch.

[21] Sato H, Teramoto S, Abe H, Mochida J, Takahashi S (2019). [LSC (laparoscopic sacrocolpopexy) versus uphold type TVM: a case control study]. Nihon Hinyokika Gakkai Zasshi.

